# Combination of Micelle Collapse and CuNi Surface Dissolution for Electrodeposition of Magnetic Freestanding Chitosan Film

**DOI:** 10.3390/nano12152629

**Published:** 2022-07-30

**Authors:** Jingyuan Bai, Meilin Zhang, Xuejiao Wang, Jin Zhang, Zhou Yang, Longyi Fan, Yanan An, Renguo Guan

**Affiliations:** 1 School of Materials Science and Engineering, Northeastern University, Shenyang 110819, China; baekkyungwon@163.com (J.B.); zhangmeilin9696@163.com (M.Z.); xuejiaowang0213@163.com (X.W.); 2 Engineering Research Center of Continuous Extrusion, Ministry of Education, Dalian Jiaotong University, Dalian 116028, China; yangzhou5959@163.com (Z.Y.); fanlongyi98@163.com (L.F.); 3 State Key Laboratory of Solidification Processing, Center of Advanced Lubrication and Seal Materials, Northwestern Polytechnical University, Xi’an 710072, China; anyanan@nwpu.edu.cn

**Keywords:** magnetic chitosan, freestanding film, CuNi nanoparticles, electrodeposition

## Abstract

Magnetic chitosan hydrogel has aroused immense attention in recent years due to their biomedical significance and magnetic responsiveness. Here, A new electrodeposition method is reported for the fabrication of a novel CuNi-based magnetic chitosan freestanding film (MCFF) in an acidic chitosan plating bath containing SDS-modified CuNi NPs. Contrary to chitosan’s anodic and cathodic deposition, which typically involves electrochemical oxidation, the synthetic process is triggered by coordination of chitosan with Cu and Ni ions in situ generated by the controlled surface dissolution of the suspended NPs with the acidic plating bath. The NPs provide not only the ions required for chitosan growth but also become entrapped during electrodeposition, thereby endowing the composite with magnetic properties. The obtained MCFF offers a wide range of features, including good mechanical strength, magnetic properties, homogeneity, and morphological transparency. Besides the fundamental interest of the synthesis itself, sufficient mechanical strength ensures that the hydrogel can be used by either peeling it off of the electrode or by directly building a complex hydrogel electrode. Its fast and easy magnetic steering, separation and recovery, large surface area, lack of secondary pollution, and strong chelating capability could lead to it finding applications as an electrochemical detector or adsorbent.

## 1. Introduction

Magnetic chitosan composites (MCCs) with significant biological and chemical properties have been of great interest during recent years in various fields of biomedical (drug delivery, artificial muscle) [1,2,3], environmental (water treatment, pollution degradation) [4,5], or even analytical (separation, biosensor) [5,6] applications. The magnetic components are usually single- or multi-magnetic cores embedded inside chitosan to ensure a strong magnetic response. Chitosan coating has proven to be useful in protecting and stabilizing magnetic cores from oxidation and aggregation [4]. Furthermore, their abundant surface functional groups (amino, hydroxyl, and carboxyl groups) can be used for other applications, such as electrochemical detection [7,8] and surface functionalization with specific components [9]. Many manufacturing process including crosslinking, precipitation, and electrodeposition are used to produce MCCs with different structures including beads, films, nanoparticles, fibers, microspheres, microcapsules, etc. [10,11,12,13,14,15,16]. Electrodeposition stands out from the rest, as it offers the advantage that assembly of stimuli-responsive chitosan can be triggered by electrical signals with exquisite spatial and temporal control [6,8,16]. With respect to the electrodeposition mechanism, electro-fabrication of chitosan can be accomplished either by cathodic neutralization or anodic deposition [8,17]. However, the structure obtained through this method is always a film or template due to the geometrical confinement induced by the electrodes.

The ability of chitosan to coordinate with transition metal ions has fostered the discovery of new electrodeposition routes and, in turn, chitosan with different morphologies can be fabricated by taking advantage of the in situ electrochemical oxidized metal ions. In this context, Wang et al. [8] demonstrated the electrodeposition method for chitosan based on coordination with metal ions generated in situ by simultaneous electrochemical oxidation. Various shapes of hydrogel coatings or films with sufficient strength can be constructed on electrodes. Nanoparticles (NPs) can also be concurrently assembled with chitosan and entrapped within the hydrogel network. In this method, the very important first step is the preparation of nanoparticles and the dispersion into chitosan solution. However, chitosan dissolves in aqueous acidic medium, which will, to some extent, corrode magnetic materials, especially if they are metal or alloys. The preparation of MCCs with other magnetic cores still remains a challenging issue. An appropriate electrodeposition route should be carefully selected in order to graft magnetic cores while avoiding or minimizing corrosion.

Concerning practical applications, an MCC must be stable, environmentally friendly, and economically viable. Therefore, iron oxides and ferrites, by far, are the most commonly used magnetic cores due to their biocompatibility, low toxicity, and chemical stability [18,19,20]. For instance, the growth of Fe_3_O_4_chitosan film has been demonstrated by cathodic codeposition from a chitosan solution containing Fe_3_O_4_ nanoparticles [6]. In fact, one-step electrodeposition is also employed to synthesize chitosan-coated Fe_3_O_4_ on the electrode, where the formation of magnetite first occurs, and then coordinates with chitosan molecules through the hydroxyl groups on the surface. In these works, MCCs are prepared in the form of films that are well-attached to the substrates, with Fe_3_O_4_ occurring as particles (micro or nano). Currently, freestanding film electrodes with favorable mechanical strength appear to be a promising candidate in the applications of lithium-ion batteries and energy storage devices [21,22,23]. They can also be directly applied as a flexible electrode for basic electrochemical characterization. However, most of the freestanding films are often prepared using different methods, such as chemical vapor deposition, template assembly, or vacuum filtration with carbon materials (e.g., graphene) as the scaffold [24,25,26]. Electrodeposition offers the possibility of fabricating polymeric freestanding film. Through careful confinement of the electric field distribution, the deposition can proceed between the gap left by the anode and cathode. However, full exploitation of electrodeposition capabilities in the field of electrofabrication of MCC freestanding film is yet to come. 

Considering the magnetic materials, the binary CuNi system has attracted the attention of researchers due to its electrocatalytic property, antifouling properties, high tensile strength, and good corrosion resistance [27,28,29,30]. From the magnetic point of view, Ni-rich CuNi alloys (i.e., Ni content greater than 61%) are known to be ferromagnetic [29]. Nevertheless, even materials rich in Cu can display ferromagnetic behavior when phase separation occurs during the preparation process. This combination of properties makes the CuNi system a promising candidate for many applications, such as thermoelectric and resistive devices or MEMS. It is worth mentioning that although the CuNi polymer composite materials have been already studied to some extent, those of the CuNi-chitosan system remain virtually unexplored. The well-dispersed CuNi nanoparticles in a chitosan matrix can not only enhance the system’s mechanical properties, but would also be a necessity for its utilization as the composite material in many applications. The electrochemically controlled deposition requires the presence of CuNi nanoparticles that are additionally added into the deposition solution before electrofabrication. Careful protection of the metallic nanoparticles is necessary in order to avoid corrosion by the acidic chitosan solution and particle agglomeration. 

In this work, CuNi-based MCC coating was prepared via electrodeposition in an acidic chitosan plating bath containing surfactant-modified CuNi nanoparticles. The modification of CuNi nanoparticles was conducted by mixing them with micelles of anionic surfactant (SDS) to protect against corrosion and particle agglomeration. CuNi can be well incorporated into the inner shell of the hydrophobic domains of the micelles. In order to maintain the pH value of the electrolyte, a relatively low applied potential was chosen to avoid hydrogen evolution. The present work aims to develop a new electrodeposition method for freestanding chitosan film through the breaking of the micelle network due to the outer energy induced by the applied potential being much greater than the association energy between micelle and the nanoparticles. This dynamic breaking process starts from the anode and proceeds towards the cathode, resulting in the exposure of the CuNi nanoparticles to the strong acidic solution. By taking advantage of the in situ generated metallic ions, chitosan hydrogel freestanding film with a smooth and homogeneous surface, and sufficient strength can be conveniently constructed. The dissolution of CuNi nanoparticles in the acidic solution will cease as long as a stable hydrogel is formed. The NPs thus have a dual function: (1) to provide the ions required for chitosan growth through their controlled surface dissolution or corrosion and (2) to endow the composite with ferromagnetic properties as they become entrapped in the chitosan matrix. Using this method, robust hydrogel with large surface area can be readily built regardless of the shape of the electrodes, which can be applied for other electrochemical analytic application afterwards. More importantly, we anticipate that other metallic nanoparticles can also be assembled with chitosan hydrogel through this method. 

## 2. Materials and Methods

### 2.1. Reagents and Materials

Chitosan (deacetylation degree, 90%), nickel (II) chloride hexahydrate (NiCl_2_·6H_2_O; 99.9%), copper (II) chloride dihydrate (CuCl_2_·2H_2_O; 99.9%), ethylene glycol (EG; 99.8%), hydrazine monohydrate (N_2_H_4_·H_2_O; 80%), SDS, and acetic acid (HAc; 98%) were purchased from Sinopharm Chemical Reagent Co., Ltd., Shanghai, China. Copper plate, platinum plate, and other chemicals were obtained from commercial sources in China. All chemicals were of analytical grade and were used as received without further purification.

### 2.2. Preparation of CuNi NPs

In a typical synthetic procedure, 1 M aqueous NiCl_2_ solution, 1 M aqueous CuCl_2_ solution, and 30 mL EG were mixed and heated to 100 °C, and then 1 mL N_2_H_4_·H_2_O was added dropwise into the mixture. The molar ratio of [Cu] to [Ni] is 2:5. This synthetic reaction was maintained at this temperature for about 30 min, followed by naturally cooling in the laboratory’s atmospheric conditions. The precipitates were collected by centrifugation and washed several times with ultrapure water and ethanol and then were dried in a vacuum oven at 40 °C for 10 h. 

### 2.3. Electrodeposition of Chitosan

Chitosan solution (1.0% *w*/*v*) was prepared by dissolving the chitosan powder in HAc (0.1 M). The pH value of the solution was then adjusted to 5.5 with diluted Hac, and careful filtering was required to remove the undissolved particles. Prior to the deposition, CuNi NPs (20 mg) were mixed with 0.02 M SDS solution and ultrasonicated for 1 h in order for the NPs to be well associated with the anionic surfactant. The electrolyte consisted of CuNi NPs-SDS suspension, 5 mL of chitosan solution, and 0.15 M NaCl. For a typical electrodeposition, two copper plates with a working area of 2.5 × 0.2 cm^2^ served as anodic and cathodic electrodes. For comparison, two platinum plates with the same working area were also used as anode and cathode. Prior to deposition, the copper and platinum plates were carefully polished and rinsed with ethanol and distilled water afterwards. Unless otherwise mentioned, the electrodeposition of chitosan freestanding film was carried out potentiostatically at *E* = −0.5 V for 40 min. The resulting chitosan film was then washed with Milli-Q water. Furthermore, instead of CuNi NPs, the graphite powder was used to electrofabricate chitosan film. The resultant film is denoted as chitosan/C. Both the anode and cathode are Cu plate, while the rest of the experimental parameters remain the same. Under the same conditions, chitosan solution without any additional NPs was used to electrodeposition, and the obtained film is denoted as chitosan/Cu^2+^ ion film. 

### 2.4. Characterization

The approximate distribution of nanoparticles in the freestanding hydrogel film was obtained using an optical microscope (Olympus BX53M, Shenzhen, China). Surface morphology and elemental analysis of the deposited freestanding hydrogel were observed using a field emission scanning electron microscope equipped with an X-ray energy dispersive system (FE-SEM, Zeiss Gemini SEM 500, Oberkochen, Germany). Fourier transform infrared spectra (FT-IR) were recorded on a Spectrum One instrument (PE, Waltham, MA, USA). For crystalline phase identification, an X-ray diffractometer in the 40–55° (step size = 0.1°, step time = 2.4 s) using Cu K_α_ radiation (λ = 0.154178 nm) was used. Room temperature hysteresis loops were collected using a vibrating sample magnetometer (VSM) from LakeShore. Electrochemical measurements were carried out on a CHI 618E electrochemical analyzer (CH Instruments, Chenhua Co., Shanghai, China). 

## 3. Results

**Figure 1:**
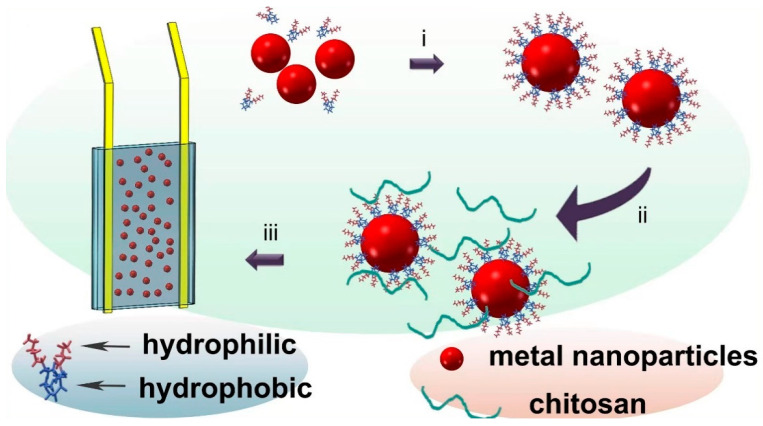
Schematic illustration of the coordinated electrodeposition of freestanding chitosan film. Step i: The surfactant and the NPs are mixed to ensure the coordination of the latter with the hydrophobic domains of the micelles. Step ii: addition of chitosan solution and stirring to trigger complexation of chitosan molecules with the hydrophilic portion of the pre-formed micelles. Step iii: electrodeposition.

**Figure 2:**
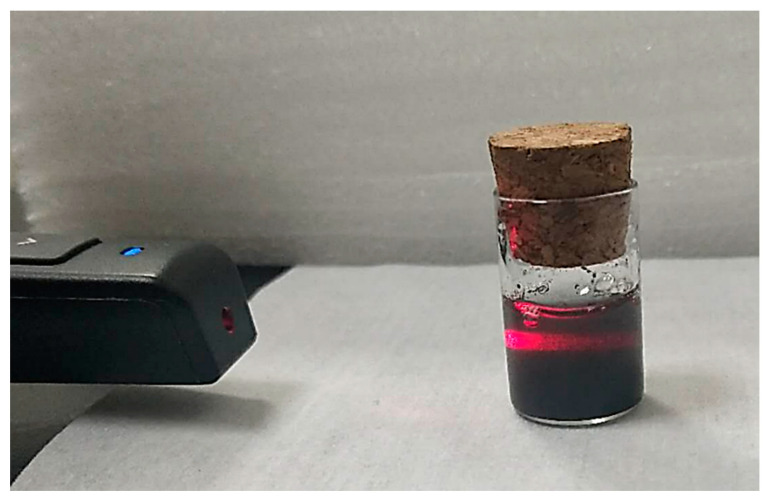
Photograph demonstrates the occurrence of Tyndall effect.

The proposed mechanism for electrodeposition of chitosan freestanding film is exhibited in Figure 1. CuNi NPs were first treated with SDS to protect them from being directly dissolved by the acidic chitosan solution. The protective capability is elucidated in terms of the physical adsorption of SDS. In this way, the alkyl chains of the adjoining SDS molecules were incorporated into the outer surface of pristine CuNi NPs, while the sulfonic groups extending into the solution provided electrostatic repulsion, maintaining a sufficient dispersion of NPs in the aqueous environment. Figure 2 exhibits a vial of NPs dispersions in SDS. The presence of micelles was proven by the so-called Tyndall effect, in which the direction of the red light beam becomes evident [31,32,33]. The SDS adsorbed at the CuNi surface is highly resistant towards corrosion by the strongly acidic chitosan solution. The chitosan solution and NaCl are added afterwards, and the pre-adsorbed SDS is bonded to chitosan due to the strong electrostatic interaction and proximity to charges carried by the surfactant head domains and the polyelectrolyte. The micelle network would collapse even under a minor applied potential due to the rather weak association energy. This dynamic breaking process starts in one electrode and proceeds toward the other one (depending on the direction of the Coulombic attraction), resulting in the exposure of the NPs to the strong acidic solution. The sodium ions at the micelle–water interface are free to dissociate from the interface, resulting in a net negative charge on each micelle. Therefore, during the electrodeposition, when a negative potential of 0.5 V is applied, the SDS micelles are attracted by the anode and then collapse, resulting in the release of the protected CuNi. Similar observations were reported by Liu, in which the SDS micelles collapsed under electric field media, releasing the protected molecules [34]. The CuNi undergoes chemical oxidation and then generates metal ions in situ. Subsequently, these metal ions can coordinate with the neighboring chitosan molecules, and then chitosan hydrogel freestanding film with a smooth and homogeneous surface and sufficient strength can be conveniently constructed. The dissolution of NPs in the acidic solution will cease as long as a stable hydrogel is formed.

**Figure 3:**
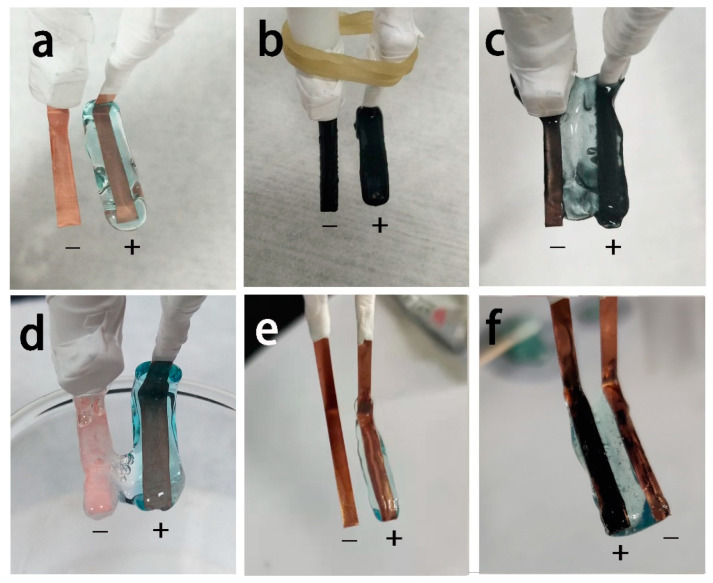
Photographs of (**a**) the chitosan/Cu^2+^ ion anodic film deposited on Cu-Cu electrodes at −0.5 V; (**b**) the chitosan/C anodic film deposited on Cu-Cu electrodes at −0.5 V; (**c**) the chitosan freestanding hydrogel film deposited on Pt-Pt electrodes at −0.5 V; (**d**) the chitosan/Cu^2+^ ion hydrogel deposited at −0.8 V; (**e**) the chitosan/Cu^2+^ ion film deposited on Cu-Cu electrode at −0.2 V; (**f**) the chitosan freestanding film deposited on Cu-Cu electrodes at −0.2 V.

A series of controlled experiments were carried out in order to further examine the key experimental factors enabling the formation of freestanding film. The experimental details and the corresponding results are illustrated in Table 1. (+, −) is used to indicate that the obtained hydrogel is freestanding film hanging in between the anode and cathode, whereas, (+) denotes that the obtained film is well attached to the anode and (×) means no reaction is detected. Firstly, it is found that the supporting electrolyte NaCl has a remarkable influence on triggering the electrodeposition. Namely, when the NaCl is absent while the rest of the conditions remain the same, no hydrogel film is observed on electrodes because the poor conductivity makes it difficult to break the micelles. In addition, in contrast to a freestanding chitosan film, a blue-colored hydrogel was deposited onto the anodic electrode (hereafter called anodic film) when CuNi NPs was missing in the electrolyte (Figure 3a). This is due to the copper plate undergoing anodic electrochemical oxidation and generating Cu^2+^ ions, which coordinate with the chitosan molecules adjacent to the anodic copper plate. This method could also be used to produce chitosan anodic film incorporated with other inorganic solids of varied structures and morphologies. For example, if inert graphite powder is used as a nanofiller instead of metallic CuNi NPs, a chitosan–graphite anodic film can be obtained under the same conditions (Figure 3b). Surface dissolution of CuNi NPs are the key factors of fabricating freestanding chitosan films. In order to prove the concept, platinum plates are also utilized as anodes and cathodes for electrodeposition, as they cannot generate metal ions that could coordinate with chitosan molecules. As shown in Figure 3c, a deposited freestanding film is observed, demonstrating that CuNi NPs has a great influence on the formation of the freestanding film. Additionally, before adding CuNi NPs into the chitosan solution, the influence of the applied potential on the freestanding film formation is also investigated. Namely, when the chitosan film was deposited at −0.8 V, the anode became oxidized and generated Cu ions in situ, forming an anodic film (Figure 3d). However, a hydrogen evolution reaction occurred on the cathode at the same time. The chitosan responded to the localized high pH value and deposited as a cathodic film. Both films were formed through different mechanisms, and thus have different physical properties and appearances. At −0.2 V, the obtained film is analogous to that of Figure 3a, in which an anodic film is observed (Figure 3e). However, at −0.2 V, after adding CuNi NPs into the electrolyte, the resultant film displays a blueish color with CuNi NPs aggregated on the anode surface. It is reasonable to conclude that at this potential value, some of the micelles collapse and expose the entrapped NPs for electrodeposition, whereas most of the micelles would migrate to the anode due to the coulombic attraction. A value of −0.5 V is suitable for the chitosan freestanding film’s deposition. The reaction ceases as long as the film connects the cathode. 

**Figure 4:**
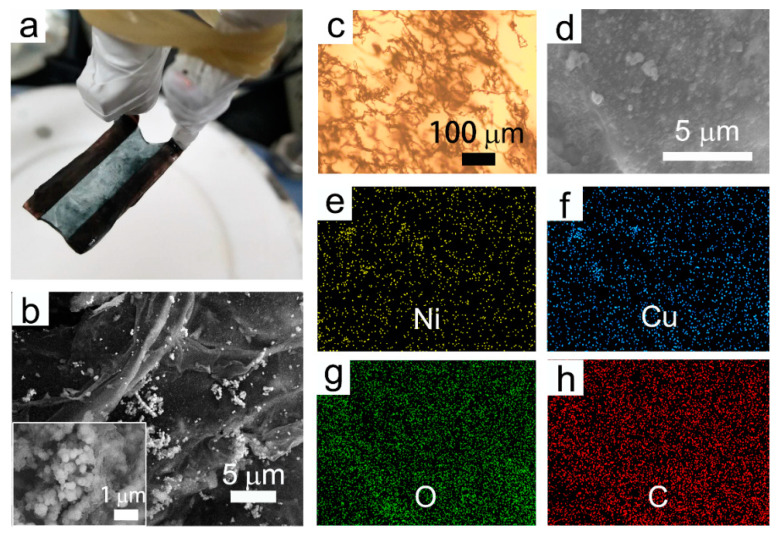
(**a**) Photograph, (**b**) SEM image, (**c**) optical microscopic image, and (**d**) zoomed SEM and the corresponding EDX mapping images (**e**–**h**) of freestanding hydrogel film deposited on the Cu electrodes (inset in (**b**) is the zoomed SEM image).

**Figure 5:**
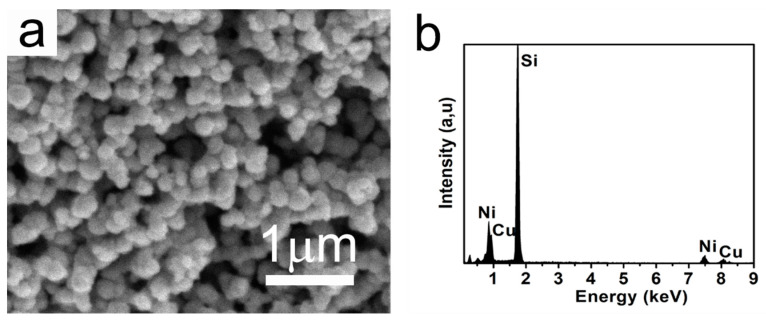
(**a**) SEM image and (**b**) EDX spectrum of the as-prepared CuNi NPs.

Some representative characterizations of the freestanding film are shown in Figure 4. There is an obvious freestanding hydrogel hanging in between the electrodes after electrodeposition, as exhibited in Figure 4a. The deposited hydrogel is transparent turquoise, which is in accordance with the typical color of Cu^2+^ and Ni^2+^ ions in aqueous solution. Additionally, it is visible that the black-colored nanoparticles render a rather uniform dispersion. In addition, this deposited freestanding hydrogel film is sufficiently robust for either peeling off from the electrodes or for future applications. Shown in Figure 4b is the SEM image, in which the discrete NPs are randomly embedded in the film. The zoomed SEM image illustrates that the CuNi NPs have sizes ranging from 90 nm to 100 nm. Furthermore, the OM (optical microscope) image in Figure 4c shows that the NPs are dispersed homogeneously over a large scale with few aggregative phenomena occurring. EDX mapping images (Figure 4d–h) show that Ni, Cu, O, and C elements co-exist in the freestanding film. The original morphology and composition of the as-prepared CuNi NPs were also investigated. As shown in Figure 5, these NPs exhibited a roughly spherical shape with a mean particle size of approximately 100–110 nm, which is slightly larger than that of the embedded particles. The corresponding EDX spectrum (see Figure 5b) shows that the composition was approximately Cu_30_Ni_70_, which is in accordance with the molar ratio of the agents added. The Si signal comes from the Si substrate onto which the NPs were drop-casted. This observation indicates that the CuNi undergoes slight surface chemical oxidation when the SDS micelles collapse, hence the reduced particle sizes. 

**Figure 6:**
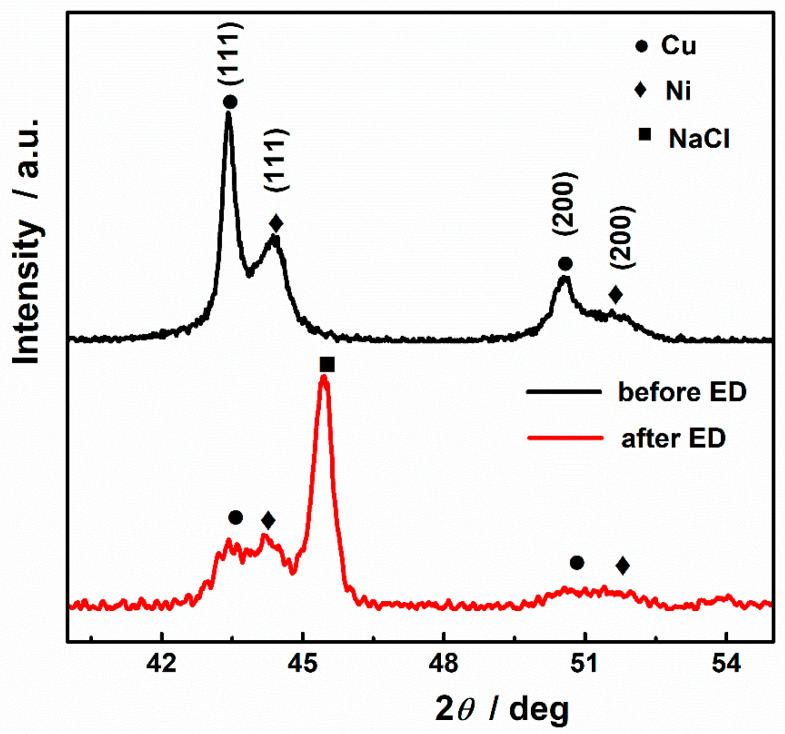
XRD patterns of the CuNi NPs (before ED) and the freestanding hydrogel (after ED) in the 40–55° 2*θ* region. Peaks denoted by ●, ◆,and ■ belong to Cu, Ni, and NaCl, respectively; ED stands for electrodeposition.

XRD analyses were carried out to investigate the crystallographic structural changes upon deposition. XRD patterns in the 40−55° 2*θ* range of the CuNi NPs and the deposited chitosan freestanding film are shown in Figure 6. The two samples show four diffraction peaks corresponding to Cu/Ni (111) and Cu/Ni (200) reflections (● and ◆, respectively), demonstrating the occurrence of two face-centered cubic structures, one rich in Cu and one in Ni. In fact, the phase separation phenomenon is quite commonly seen during the preparation procedure for the CuNi system. Our group recently prepared CuNi via electrodeposition and chemical coreduction [28,30], both of which produced nanomaterials consisting of Cu-rich and Ni-rich phases. The different reduction potentials and nucleation rates are responsible for the phase separation. Moreover, the total free energy of the system is lower when Ni atoms segregate to form Ni-rich magnetic clusters on the basis of the local environment model [35,36]. By comparing the XRD pattern of CuNi and chitosan freestanding film, all the peaks corresponding to Cu- and Ni-rich phases can be indexed. However, they also present significant changes in both relative peak intensity and width, indicating that the CuNi NPs are involved in electrodeposition. Rietveld quantitative phase analysis of XRD patterns was implemented using GSAS software. The amounts of the Cu-rich and Ni-rich phases were estimated to be 29.6% and 70.4%, respectively, for CuNi NPs and 41.7% and 59.3%, respectively, for chitosan freestanding film. The precise stoichiometry of the Cu-rich and Ni-rich phases is given in Table 2. As Cu is a more noble metal than Ni, phases with higher Cu content are more corrosion-resistant and thus the little changes in composition. The corrosion takes place mainly on the Ni-rich domain, and the Ni content in the Ni-rich phase decreases from 99.24% to 95.88%. A new peak appears at 45.65° and can be indexed to NaCl with the reference code (00-005-0628). This can be attributed to the recrystallization of NaCl in hydrogel which, suffered slightly from dehydration during characterization. 

**Figure 7:**
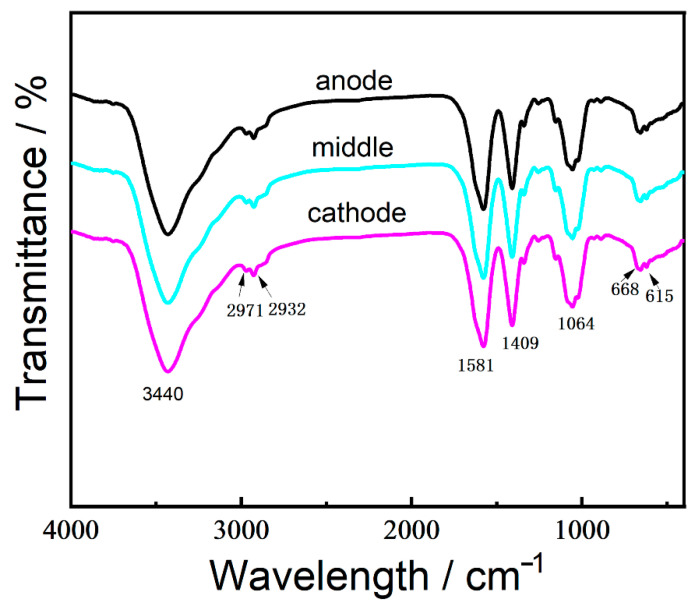
FTIR spectra of three different positions of the freestanding hydrogel film (anodic, middle, and cathodic film).

In order to confirm the homogeneity of the freestanding chitosan film, specimens taken from the position close to the anode, cathode, and the middle section were subjected to FTIR analyses (Figure 7). In general, the shape of three curves were analogous to each other and similar to that of the intermediate hydrogel, indicating that the uniformity of the as-prepared hydrogel film. The peak observed at around 3440 cm^−1^ relates to O-H and N-H stretching vibration. It is also observed that bands at 2971 and 2932 cm^−1^ are the characteristic peaks of C-H stretching. The peak at 1581 cm^−1^ can be assigned to the bending vibration of NH_2_ in amine, and the peak appearing at 1409 cm^−1^ is the C-N stretching vibration. The peaks for C-O stretching in the monosaccharide rings and C-O-H groups merge into a broad band at 1064 cm^−1^. Moreover, the FTIR spectra also show two peaks at 615 and 668 cm^−1^, which are associated with the stretching modes of S-O in the SDS molecules. 

**Figure 8:**
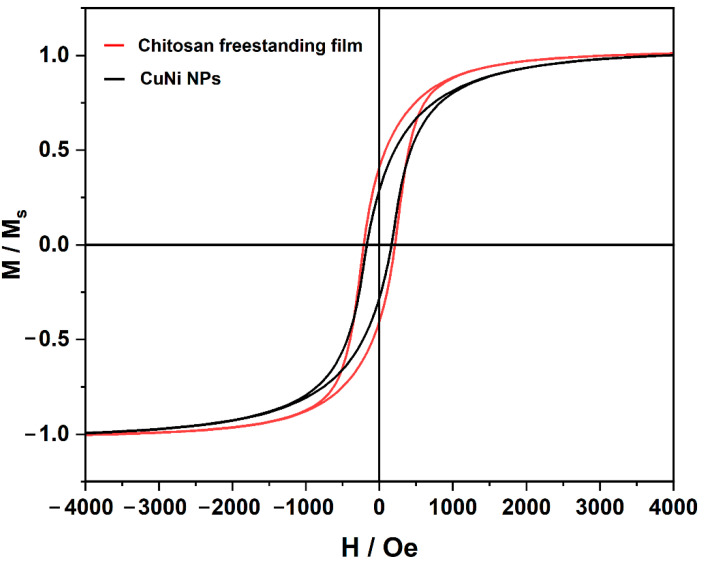
Room-temperature hysteresis loops of CuNi NPs and chitosan freestanding film.

**Figure 9:**
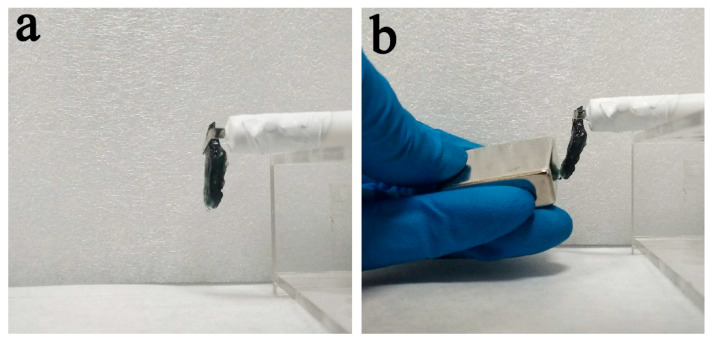
Chitosan film (**a**) hanging on electrode, (**b**) being attracted by a magnet.

Room-temperature magnetic hysteresis loops of the CuNiNPs and the freestanding hydrogel are shown in Figure 8. The saturation magnetization (*M*_s_) for the chitosan freestanding hydrogel film (167 Oe) is obviously lower than that of CuNi NPs (226 Oe). Ni content decreases along with the chemical oxidation, causing the decline of *M*_s_. The decrease in *M*_s_ indicates that Ni is involved in the chitosan freestanding film’s deposition. The hysteresis loops also reflect that the coercivity (*H*_c_) values of freestanding hydrogel (around 170 Oe) are smaller than those of CuNi NPs (around 226 Oe). The reduction of coercivity can be considered in many aspects. On one hand, Figure 6 reveals that the width of the XRD peaks of Ni and Cu become wider after electrodeposition, which indicates that the crystallite sizes of Ni and Cu decrease. Generally, grain boundaries block and pin the propagation of magnetic domain walls [37]. *H_c_*, being inversely proportional to the grain size, will increase as a result [38]. On the other hand, the distance between the neighboring nanoparticles in the synthesized hydrogel film becomes greater compared to that of the CuNi NPs. The weakened dipolar interactions decrease the cooperative reversal of the NPs and tend to decrease *H_c_*. Moreover, the chitosan freestanding hydrogel film obtained by this method has certain magnetic responsiveness and sufficient mechanical robustness (see Figure 9). The hydrogel film remains in its original shape after peeling off from the electrode, and it can be attracted towards the magnet. Therefore, these materials could find applications such as wastewater remediation, in which the abundant surface functional groups on chitosan will target pollutant removal, while the CuNi NPs in the interior will ensure magnetic guidance and recovery. 

## 4. Conclusions

We report a novel electrodeposition method of CuNi-chitosan freestanding film based on micelle collapse and coordination of chitosan with the metal ions generated in situ by concurrent chemical oxidation. In particular, this novel synthetic process is triggered by the coordination of chitosan with metal ions generated in situ by the controlled CuNi surface dissolution of the suspended metallic NPs with the acidic plating bath. The entrapped CuNi NPs have a Cu-rich phase and a Ni-rich phase. Due to the Cu-rich phase being more corrosion-resistant, the Ni-rich phase is more involved in the chitosan coordination process. The obtained chitosan freestanding film has smooth and homogeneous morphology, magnetic properties, and adequate strength to be peeled off of the electrodes. Using this method, robust MCFF with a variety of shapes and large surface area can be readily built up. 

## Figures and Tables

**Table 1:** Electrodeposition details and the corresponding result. (√) indicates the utilization of the referred chemicals, (+) indicates the anodic film, (×) indicates no reaction, (+, −) indicates the freestanding film.

Electrolyte					Electrode		Results
Chitosan	NaCl	SDS	CuNi NPs	Graphite Power	Anode	Cathode	
√	√	√	√		Cu	Cu	+, −
√		√	√		Cu	Cu	×
√	√	√			Cu	Cu	+
√	√	√		√	Cu	Cu	+
√	√	√	√		Pt	Pt	+, −

**Table 2:** Composition of the Ni-rich and Cu-rich phases present in the CuNi NPs and chitosan freestanding film, as determined from XRD analyses.

	Ni-Rich Phase	Cu-Rich Phase
**CuNi NPs**	2*θ* = 44.46 (deg)	2*θ* = 43.47 (deg)
	d = 2.036	d = 2.080
	a = b = c = 3.527 Å	a = b = c = 3.603 Å
	at% Ni = 99.24%	at% Cu = 94.27%
	at% Cu = 0.76%	at% Ni = 5.73%
**Chitosan freestanding film**	2*θ* = 43.58 (deg)	2*θ* = 42.60 (deg)
	d = 2.075	d = 2.120
	a = b = c = 3.595 Å	a = b = c = 3.671 Å
	at% Ni = 95.88%	at% Cu = 94.47%
	at% Cu = 4.12%	at% Ni = 5.53%

## Data Availability

The data presented in this study are available on request from the corresponding author.
